# Burn wound healing property of *Cocos nucifera*: An appraisal

**DOI:** 10.4103/0253-7613.43159

**Published:** 2008-08

**Authors:** Pallavi Srivastava, S. Durgaprasad

**Affiliations:** Department of Pharmacology, KMC International Centre, Manipal, India

**Keywords:** *Cocos nucifera*, epithelialization, silver sulphadiazine, wound contraction

## Abstract

**Objectives:**

The study was undertaken to evaluate the burn wound healing property of oil of *Cocos nucifera*  and to compare the effect of the combination of oil of *Cocos nucifera*  and silver sulphadiazine with silver sulphadiazine alone.

**Materials and Methods:**

Partial thickness burn wounds were inflicted upon four groups of six rats each. Group I was assigned as control, Group II received the standard silver sulphadiazine. Group III was given pure oil of *Cocos nucifera* , and Group IV received the combination of the oil and the standard. The parameters observed were epithelialization period and percentage of wound contraction.

**Results:**

It was noted that there was significant improvement in burn wound contraction in the group treated with the combination of *Cocos nucifera*  and silver sulphadiazine. The period of epithelialization also decreased significantly in groups III and IV.

**Conclusion:**

It is concluded that oil of *Cocos nucifera* is an effective burn wound healing agent.

## Introduction

Burn can be defined as tissue damage caused by a variety of agents such as heat, chemicals, electricity, sunlight, or nuclear radiation. The most common are burns caused by scalds, building fires, and flammable liquids and gases. Every year, about two million people receive medical treatment for burn injury.[1] Thermal burn and related injuries have remained a major cause of death and disability.[2] Although small burns are not usually life threatening, they need the same attention as large burns, in order to achieve functional and cosmetic outcome.[3]

Most of the early treatment modalities include topical application of medicament, mainly aimed at preventing infections. Improving the methods of wound healing and tissue repair offers tremendous opportunities to enhance the quality of life for trauma and burn patients. It may also help to reduce health care costs. Many of the drugs used today have their origin in plant medicine.[4]

*Cocos nucifera*  Linn (Arecaceae) is a stately palm, which thrives within the tropical zone. Commonly known as coconut, it is known as Narikela in Sanskrit. The most important coconut producing countries in the world are India, Sri Lanka, Malaysia and Indonesia. Coconut is widely used in Asian countries for a variety of purposes. Its fresh kernel is consumed by people all over India and the kernel forms an ingredient of many Indian food preparations. Oil of *Cocos nucifera*  (CN) is extracted from copra, which is the dried inner flesh of coconut. Coconut oil consists of about 90% saturated fat. The oil contains predominantly medium chain triglycerides, with 86.5% saturated fatty acids, 5.8% monounsaturated fatty acids, and 1.8% polyunsaturated fatty acids. Of the saturated fatty acids, coconut oil is primarily 44.6% lauric acid, 16.8% myristic acid and 8.2% palmitic acid, although it contains seven different saturated fatty acids in total. Its only monounsaturated fatty acid is oleic acid, while its only polyunsaturated fatty acid is linoleic acid.[5] Virgin coconut oil has beneficiary effect in lowering serum lipid levels.[6] Lauric acid present in the oil can kill some bacteria.[7] It is also known to have antibacterial and antifungal activity.[8] A study shows that extra virgin coconut oil is as effective and safe as mineral oil when used as a moisturizer, with absence of adverse reactions.[9] In the present study, we have examined the effect of CN oil on artificially inflicted burn wounds in rats.

## Materials and Methods

### Animals

Twelve-week-old healthy Wistar rats weighing 150-200 g of either sex, bred locally in the animal house of Kasturba Medical College, Manipal, were selected for the study. They were housed under controlled conditions of temperature (23 ± 2°C), humidity (50 ± 5%) and 10-14 hours of light and dark cycles. The animals were housed individually in polypropylene cages containing sterile paddy husk bedding and free access to food and water *ad libitum*.

The study was conducted after obtaining the approval of the Institutional Animal Ethics Committee.

### Study design

The animals were randomly allocated into four groups of six animals each. 

Group I was assigned as control.

Group II received the standard – silver sulphadiazine cream (SSD) 0.5 g of 1% daily

Group III received pure oil of CN 1 ml. 

Group IV received both oil of CN and the standard SSD cream.

### Dosing schedule

Oil of CN and SSD were administered topically, once daily from day 0 to the day of complete healing or the 21^st^  postoperative day, whichever occurred earlier, in the partial thickness burn wound model.[10]

### Wound model

Partial thickness burn wounds were inflicted upon animals starved overnight and under pentobarbitone anesthesia, by pouring hot molten wax at 80°C into a metal cylinder with 300 mm^2^ circular opening, placed on the back of the animal.[11]

The parameters observed in the study were as follows:

- Epithelialization period: It was monitored by noting the number of days required for the eschar to fall off from the burn wound surface without leaving a raw wound behind.
- Wound contraction: It was noted by following the progressive changes in wound area planimetrically, excluding the day of the wounding. The size of the wounds was traced on a transparent paper every two days, throughout the monitoring period. The tracing was then transferred to 1 mm^2 ^ graph sheet, from which the wound surface area was evaluated. The evaluated surface area was then employed to calculate the percentage of wound contraction, taking the initial size of the wound, 300 mm^2^ , as 100%, by using the following equation:
  $$\text{Percentage of wound contraction}=\frac{\text{Initial wound size}-\text{specific day wound size}}{\text{Initial wound size}}\times 100$$

### Statistical analysis

The results were analyzed using One-way ANOVA followed by *post hoc*  test viz Tamhane's.

## Results

The mean period of epithelialization was found to decrease significantly in SSD treated group (*P*  < 0.001). In the combination group, it was not statistically significant [Table 1]. The percentage of burn wound contraction in the CN treated group was found to increase on the 8^th^  day onwards. On the 12^th^  day, the percentage of wound contraction was found to increase significantly in the standard group (73.2 ± 5.9, *P*  < 0.001) and in the rats treated with the combination (82.1 ± 7.2, *P*  < 0.001). On the 16^th^  day, the percentage of wound contraction was found to increase in all the three drug treated groups. 

**Table 1:** Effect of Cocos nucifera on wound contraction and epithelialization period

*Treatment (N=6)*		*% of wound contraction (mean±SE)*				*Period of epithelialization in days (mean±SE)*
			
		*4_th_ day*	*8_th_ day*	*12_th_ day*	*16_th_ day*	
Control		15.1±3.1	19.1±3.3	27.6±4.3	34.9±2.4	54.6±1.5
SSD		10.2±4.5	29.8±3.2	73.2±5.9^b^	95.5±4.4^b^	29.0±1.4^b^
CN		10.7±0.6	14.2±1.5^a^	49.2±7.7	84.7±6.8^b^	39.3±4.1
SSD+CN		10.1±4.2	43.1±12.6	82.1±7.2^b^	92.1±4.1^b^	39.6±6.1
One-way					
ANOVA	F	2.487	3.568	14.349	35.352	7.594
	P	<0.090	<0.032	<0.001	<0.001	<0.001

SSD= Silver sulphadiazine, CN= Cocos nucifera

a P <0.020 against SSD

b P <0.001 against control

## Discussion

The present study shows a significant improvement in burn wound contraction in the rats treated with CN and the combination of CN and SSD. Since the combination of CN and SSD significantly influenced the process of burn wound healing, it can be said that oil of *Cocos nucifera*  could be a cheap and effective adjuvant to other topical agents, for attaining faster healing of wounds, without complications. In the past, many studies have been done using natural products for the treatment of burn wounds, but these were mainly aimed at controlling infections.[12,13] Anti-inflammatory activity of certain natural products could also play a part in the healing of burn wound.[14] Although the present study did not explore the exact mechanism of prohealing of CN, it could be attributed to both anti-inflammatory and antiseptic properties. A clinical study showed that in the treatment of superficial and deep second-degree burns, addition of another prohealing agent like hyaluronic acid significantly overcame the disadvantages of silver sulphadiazine.[15] Based on these, we propose that oil of *Cocos nucifera*  could significantly enrich the assortment of topical medications available for the treatment of burns.
